# Sudden anaerobization in *Amphibacillus xylanus* increases intracellular labile ferrous iron and inhibits cell growth

**DOI:** 10.1002/2211-5463.70255

**Published:** 2026-04-27

**Authors:** Shinya Kimata, Yoichi Sakai, Keisuke Tanaka, Etsuro Yoshimura, Ken Kitano, Morio Ishikawa, Tadahiro Nakamoto, Tsutomu Takayama, Hirofumi Inoue, Kaho Nomura, Tomonori Suzuki, Masataka Uchino, Akira Abe, Youichi Niimura

**Affiliations:** ^1^ Department of Molecular Microbiology Tokyo University of Agriculture Japan; ^2^ Department of Chemistry Daido University Nagoya Japan; ^3^ Department of Informatics Tokyo University of Information Sciences Chiba Japan; ^4^ Department of Applied Biological Chemistry The University of Tokyo Japan; ^5^ Division of Biological Science Nara Institute of Science and Technology Japan; ^6^ Department of Fermentation Science Tokyo University of Agriculture Japan; ^7^ Department of Materials Characterization Toray Research Center, Inc. Otsu Japan; ^8^ Department of Nutritional Science and Food Safety Tokyo University of Agriculture Japan; ^9^ Division of Nephrology, Department of Internal Medicine University of Michigan Ann Arbor MI USA

**Keywords:** ^57^Fe Mössbauer spectroscopy, free flavin, labile ferrous iron

## Abstract

*Amphibacillus xylanus* is an aerotolerant anaerobe that consumes large amounts of oxygen and requires iron as an essential micronutrient during its aerobic growth. However, this bacterium lacks a typical respiratory chain; therefore, intracellular free flavins and their reductases are thought to participate in the reduction of molecular oxygen and ferric iron (Fe^3+^). This system can potentially generate hydroxyl radicals through the Fenton reaction, highlighting the importance of regulating the production of redox‐active ferrous iron (Fe^2+^). In this study, we employed a strategy of abruptly switching the cell culture system from aerobic to anaerobic conditions to examine Fe^2+^ production via free flavins. Sudden anaerobization induced growth inhibition and a significant increase in intracellular labile Fe^2+^. Whole‐cell ^57^Fe Mössbauer spectroscopy revealed that in aerobic cells, high‐spin Fe^3+^ is the major chemical species, whereas in anaerobic conditions, the intracellular iron pool is completely converted into Fe^2+^ and consists of low‐ and high‐spin Fe^2+^ species. Increased Fe^2+^ production was mimicked using cell‐free extracts and the reductase activity promoted electron transfer from NADH to Fe^3+^ via physiological concentrations of free flavin adenine dinucleotide under anaerobic conditions. RNA sequencing showed that the electrons of NADH generated through glycolysis and the pyruvate metabolic pathway can flow into a flavoprotein with flavin reductase activity. These findings suggest that the production of labile Fe^2+^ via free flavin is regulated by molecular oxygen, allowing safe iron utilization during the aerobic growth of *A. xylanus*.

## Graphical abstract

Abruptly changing from aerobic to anaerobic conditions (sudden anaerobization) induced growth inhibition and a significant increase in intracellular labile ferrous iron in the aerotolerant anaerobe *Amphibacillus xylanus*. We found that free flavins mediate efficient electron transfer from NADH to ferric iron under anaerobic conditions, suggesting that molecular oxygen regulates labile ferrous iron production via free flavins in *A. xylanus*.

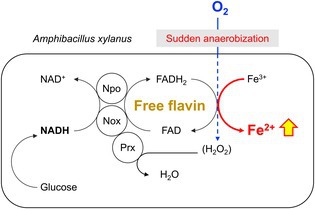

**Abbreviations**
CFE: cell‐free extract
FAD: flavin adenine dinucleotide
FMN: flavin mononucleotide
Nox: NADH oxidase
Npo: NAD(P)H oxidoreductase
Prx: peroxiredoxin

*Amphibacillus xylanus* (*A. xylanus*) is an aerotolerant anaerobic bacterium capable of assimilating xylan under alkaline conditions, regardless of oxygen, making it a promising candidate for biomass utilization [1, 2]. *A. xylanus* grows well in both aerobic and anaerobic environments, with similar growth rates and cell yields [3]. This is due to the presence of a glycolytic metabolic pathway and both aerobic and anaerobic pyruvate metabolic pathways that produce comparable amounts of adenosine triphosphate (ATP) under each growth condition [4]. In static liquid cultures, this bacterium rapidly consumes dissolved oxygen and establishes an anaerobic environment suitable for xylanase activity [1]. Although the oxygen consumption rate of *A. xylanus* is comparable to that of *Escherichia coli*, it lacks a respiratory chain that serves as the central oxygen metabolic system in *E. coli* [5]. We found that *A. xylanus* has an alternative system that utilizes large amounts of oxygen, including free flavins [5] (Fig. 1).

**Fig. 1:**
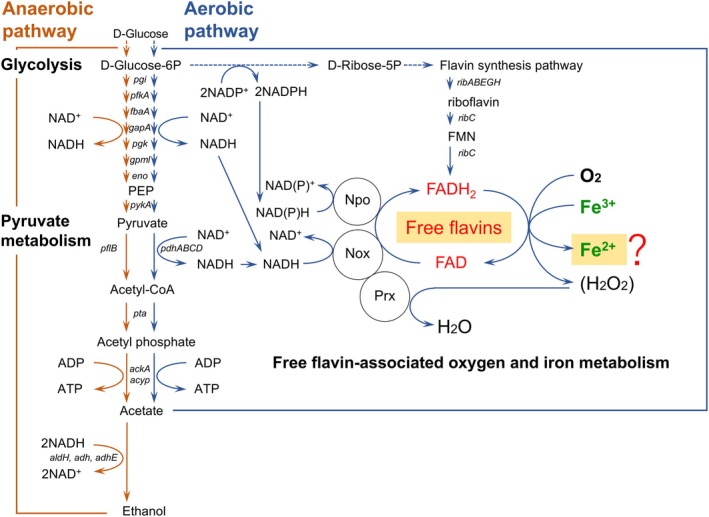
The central metabolic pathway for aerobic and anaerobic growth in *Amphibacillus xylanus. A. xylanus* uses glycolysis and two different pyruvate metabolic pathways for aerobic and anaerobic growth as its central energy metabolism. Free flavin and its associated enzymes participate in oxygen and iron metabolism and maintain the redox balance under aerobic conditions.

Flavins are generally present in cells as flavin adenine dinucleotide (FAD) or flavin mononucleotide (FMN), bound to proteins, and involved in redox reactions. However, free flavins are not bound to proteins, but undergo one‐ or two‐electron redox reactions. Free reduced flavins can reduce molecular oxygen (O_2_) to hydrogen peroxide (H_2_O_2_) or ferric iron (Fe^3+^) to ferrous iron (Fe^2+^) owing to their low redox potential [6, 7, 8]. Free reduced flavins are generated through flavin reductase activity and catalyzed by NAD(P)H‐dependent flavin reductases. These enzymes have been identified in several microorganisms as H_2_O_2_‐forming NAD(P)H oxidases [5, 9, 10, 11] or ferric reductases [12, 13, 14, 15, 16, 17, 18, 19, 20, 21, 22]. *A. xylanus* contains two flavoproteins that function as flavin reductases, FAD‐binding NADH oxidase (Nox) and FMN‐binding NAD(P)H oxidoreductase (Npo). Their O_2_ reductase activity dramatically increased with increasing physiological concentrations of free FAD [5]. Because these activities effectively re‐oxidize NAD(P)H, we hypothesized that free flavins and their associated enzymes are involved in maintaining the redox balance during the aerobic growth of *A. xylanus*. This is a unique case in which free flavins are involved in the central metabolism of glucose (glycolysis and pyruvate metabolism) (Fig. 1).

This system also participates in iron metabolism. *A. xylanus* requires iron as an essential metal for aerobic growth, and iron is used as a cofactor for iron‐binding proteins [5]. Apoproteins are thought to adsorb iron in the form of ferrous ions. However, Fe^2+^ is easily oxidized under aerobic conditions and becomes insoluble. Therefore, the reduction of ferric iron to Fe^2+^ is important for iron utilization, and one study has suggested that *A. xylanus* relies on free flavins for efficient Fe^2+^ acquisition [5].

However, if free reduced flavins simultaneously produce H_2_O_2_ and Fe^2+^ in *A. xylanus* cells, these species react with each other to generate hydroxyl radicals via the Fenton reaction, thereby promoting oxidative damage. Hydroxyl radicals are highly reactive oxygen species (ROS) that attack most of organic molecules such as lipids, proteins, and nucleic acids [23]. Therefore, *A. xylanus* must maintain H_2_O_2_ and Fe^2+^ below toxic levels to prevent the Fenton reaction. Moreover, H_2_O_2_ is rapidly reduced to water by the NADH oxidase‐alkyl hydroperoxide reductase C system (Nox‐Prx), which enables *A. xylanus* to adapt to forced oxidative stress conditions, such as exposure to 80% O_2_ or 0.1 mm H_2_O_2_ [24, 25, 26], even sudden exposure to 21% oxygen (Fig. S1). However, whether free reduced flavins generate Fe^2+^ in *A. xylanus* cells, particularly in unstable forms, remains unclear (Fig. 1). Labile Fe^2+^ is considered redox‐active and is involved in biological processes involving both iron utilization and toxic radical generation.

In this study, we aimed to investigate the production of labile Fe^2+^ via free reduced flavins in *A. xylanus*. However, detecting intracellular Fe^2+^ during aerobic growth is difficult because Fe^2+^ is easily oxidized by oxygen. To detect intracellular Fe^2+^, the growth environment was abruptly switched from aerobic to anaerobic; we defined this immediate environmental switch as ‘sudden anaerobization’ in this study. This study suggests that intracellular free reduced flavins in *A. xylanus* can increase labile Fe^2+^ levels upon sudden anaerobization.

## Methods

### Bacterial culture

*Amphibacillus xylanus* Ep01, which was isolated from an alkaline fermented compost [1], was grown in alkaline semi‐defined medium (pH 9.7) at 39.5 °C [5]. However, L (+)‐ascorbic acid sodium salt was excluded from the regular medium to avoid the unintended reduction of Fe^3+^ when assessing intracellular Fe^2+^. The composition of the alkaline semi‐defined medium was as follows (L^−1^): 2 g ammonium nitrate, 1 g K_2_HPO_4_, 3.75 g casamino acid, 20 mg tryptophan, 0.2 mg biotin (+), 0.2 mg folic acid, 1 mg pyridoxine hydrochloride, 0.5 mg riboflavin, 0.5 mg thiamine hydrochloride, 0.5 mg nicotinic acid, 0.5 mg calcium (+)‐pantothenate, 0.1 mg cyanocobalamin, 0.5 mg *p*‐aminobenzoic acid, 0.25 mg orotic acid monohydrate, 0.25 mg thymidine, 0.25 mg inosine, 0.1 mg (±)‐thioctic acid, 10 mg adenine, 10 mg guanine hydrochloride, 10 mg uracil, 10 mg xanthine, 50 mg L(−)‐cysteine, 0.2 g MgSO_4_·7H_2_O, 5 mg MnSO_4_·5H_2_O, 5 mg FeSO_4_·7H_2_O, 5 mg ZnSO_4_·7H_2_O, 0.1 g CaCl_2_·2H_2_O, 0.5 mg CuSO_4_·5H_2_O, 1 mg resazurin sodium salt, 120 mL 25% D(+)‐glucose, 100 mL 1 M NaHCO_3_‐NaH_2_CO_3_ (pH 10.6), and 780 mL ultrapure water. Initially, aerobic culture was initiated with an optical density of 660 (OD_660_) of approximately 0.1 in a jar fermenter (MDL‐1001S; B.E. Marubishi, Tokyo, Japan) containing 4 L of medium supplied with air (21% O_2_/79% N_2_‐mixed gas) at 0.5 volume/volume/min and agitated at 100 rpm. When the OD_660_ reached approximately 1.0, 1 L of culture was sampled to obtain aerobically cultured cells, and 100% N_2_ gas was supplied to the remaining culture in the jar immediately after stopping the air supply to change into anaerobic conditions. Subsequently, glucose solution was supplemented at a final concentration of 1% as the growth substrate for additional anaerobic culture. The complete change from aerobic to anaerobic conditions was confirmed by monitoring dissolved oxygen using a Clark‐type oxygen electrode. The anaerobically cultured cells were harvested 30 and 240 min after the N_2_ gas supply was initiated. Cell pellets obtained by centrifugation at 12 000× *g* for 5 min at 4 °C were washed three times with 50 mm HEPES‐NaOH buffer (pH 7.0) and stored at −80 °C until use for evaluation of intracellular free flavin and enzymatic activity.

### Viable bacterial cell count

The number of viable cells was measured on modified alkaline semi‐defined medium plate that contained 1% glucose, 50 mm of NaHCO_3_‐Na2CO_3_ (pH 9.7), and 1.8% agar after incubation aerobically or anaerobically at 39.5 °C for 18–24 h. Student's paired *t*‐test was used to compare the differences between sample types, and a *P‐*value < 0.05 indicated a significant difference.

### RNA sequencing (RNA‐seq) analysis

RNA was isolated from the cultured cells using an RNeasy mini kit (QIAGEN, Hilden, Germany). Quality was evaluated based on the RNA integrity number using the Agilent RNA 6000 Nano Kit (Agilent Technologies, Inc., Santa Clara, CA, USA) and was accepted if the value was > 7. Ribosomal RNA was removed from the total RNA samples using the NEBNext rRNA Depletion Kit (Bacteria) prior to the construction of complementary DNA library using the NEBNext Ultra II RNA Library Prep Kit for Illumina. The library was sequenced on an Illumina NextSeq 500 platform to generate 2 × 75‐bp paired‐end reads. The read data were submitted to the DDBJ Read Archive (accession number PRJDB35440). Sequencing data were analyzed using CLC Genomics Workbench software (QIAGEN, Hilden, Germany). Adapter sequences in each read were trimmed, and the cleaned reads were mapped to the reference genome of *Amphibacillus xylanus* (RefSeq assembly accession: GCF_000307165.1) to evaluate gene expression. The data were normalized using the transcripts per million (TPM) method. We defined as differentially expressed genes (DEGs) as any gene showing a fold change value of ≥ |2| and false discovery rate (FDR) *P*‐value of < 0.05.

### Preparation of cell‐free extract

The cells were resuspended in four volumes of ice‐cold 50 mm HEPES‐NaOH buffer (pH 7.0) and homogenized by passing through a French pressure cell (FA078A from SLM‐AMINCO; Thermo Fisher Scientific, Inc., Waltham, MA, USA) three times at 140 MPa and subsequently placed on ice for cooling. The extraction was centrifuged at 12 000× *g* for 20 min at 4 °C, and the supernatant was collected as cell‐free extract (CFE).

### Quantification of intracellular free flavins

Intracellular free flavins were measured as described previously [5], and free flavins were fractionated from the CFE using centrifugal membranes that exclude proteins larger than 3 kDa. The fractionated flavin samples were analyzed using a high‐performance liquid chromatography (HPLC) system with an ODS column that detected flavins at excitation and emission wavelengths of 450 and 530 nm, respectively. A standard sample containing FAD, FMN, and riboflavin was analyzed to generate calibration curves. The resulting flavin content values were converted to intracellular concentrations based on the weight ratio of wet‐to‐dry cells. Wet and dry cell weights were determined as previously described [5].

### Free flavin‐associated ferric reductase activity

Ferric reductase activity of CFEs was measured anaerobically or aerobically in a reaction mixture that contained 50 mm sodium phosphate (pH 7.0), 150 μm NADH, 100 μm ferric citrate, 400 μm ferrozine, and various concentrations of FAD (0–7 μm) at 25 °C. The reaction was initiated by adding CFE containing 200 μg of protein. Formation of a purple‐colored ferrous iron‐ferrozine complex was monitored by measuring the increase in absorbance at 562 nm using a spectrophotometer (U‐3900H, Hitachi, Ltd., Tokyo, Japan).

### Imaging of intracellular ferrous iron

*Amphibacillus xylanus* was cultured in a jar fermenter using the method described above. The intracellular ferrous iron in the cells was reacted with 5 μm FeRhoNox‐1 reagent (Goryo Chemical, Inc., Hokkaido, Japan) at 30 °C for 1 h with shaking at 120 rpm in the dark. This reaction was conducted aerobically for the aerobic cells and anaerobically in a vial filled with 100% N_2_ gas for the anaerobic cells. After incubation, the cells were collected after centrifugation at 20 000× *g* for 1 min at 4 °C and washed three times with 20 mM sodium phosphate buffer (pH 7.0). The cells were resuspended with the same buffer and diluted to an OD_660_ of 0.2–0.3. The sample (5 μL) was dropped onto a black Teflon printed microscopic slide and air‐dried in the dark. The cells were analyzed under a BX53 fluorescence microscope (Olympus, Tokyo, Japan). Intracellular FeRhoNox‐1 exhibiting red fluorescence was detected using a green excitation filter, and the images were captured using the cellSens software (Olympus, Japan). For each sample type, 15 field images were obtained from three biological experiments and analyzed using the imagej software (version 1.15 m9) [27] to determine the average fluorescence intensity per cell. Student's paired *t*‐test was used to compare the differences between sample types and *P‐*value < 0.05 indicated a significant difference.

### Measurement of intracellular total iron content

The freshly cultured cells were washed three times with 50 mm HEPES‐NaOH buffer (pH 7.0) and pelleted by centrifugation at 48380× *g* for 20 min at 4 °C. The cell pellets were then dried at 105 °C until their weights no longer changed. The dried cells were transferred to a Teflon vessel containing 2 mL of 3 N HNO_3_, and the vessel was tightly shielded in a metal jacket. The metals in the samples were dissolved in a dry heater by gradually increasing the heating temperature initially to 100 °C for 30 min, followed by 120 °C for 30 min, 140 °C for 30 min, and finally 150 °C for 6 h. After cooling to room temperature, the samples were collected and 5 mL of 0.1 N HNO_3_ were added. The iron content was assayed using the Nitroso‐PSAP method [28]. Intracellular concentrations of iron were estimated based on the weight ratio of wet‐to‐dry cells.

### ^57^Fe‐Mössbauer spectroscopy

*Amphibacillus xylanus* was grown in ^57^Fe‐enriched semi‐defined medium. FeSO_4_·7H_2_O was omitted from each regular medium and ^57^Fe (III)‐oxide was added instead. The supplemented ^57^Fe (III) citrate solution was prepared as follows: Fe (III)‐oxide containing approximately 95% ^57^Fe isotopes was dissolved in 12 N HCl by heating, diluted 10 times with ultrapure water, and then six molar equivalents of trisodium citrate dihydrate were added and adjusted to pH 6–7 with NaOH until the solution became clear. The concentration of iron in the solution was determined using a bathophenanthroline assay and the solution was diluted to 100 ppm. The solution was added to the medium at a final concentration of 1 ppm for bacterial growth. The cultured cells were harvested by centrifugation at 12 000× *g* for 5 min at 4 °C, and the cell pellet was immediately transferred into a lead holder (2‐mm thick) and frozen on dry ice. For the anaerobically cultured cells, an anaerobic glove box was used to prevent iron oxidation. The thin pellet was wrapped and shielded with polyethylene film and stored at −80 °C until Mössbauer measurements. Mössbauer measurements were performed at 78 K in conventional transmission mode on a Mössbauer spectrometer (Model‐222B from Toporogic System Co.) with a ^57^Co (Rh) source (925 MBq). Spectral curve fitting was performed using MossWinn software (version 4.0), based on the assumption of a linear combination of the Lorentzian curves. From the spectral deconvolution, we evaluated Mossbauer parameters such as isomer shifts (IS, *δ*) and quadrupole splittings (QS, *ΔE*_*Q*_). Doppler velocity scale was calibrated with respect to α‐iron at room temperature, and IS was measured relative to α‐iron at room temperature. Our assignment of Fe species was based on the IS‐QS relationship presented by Murad and Cashion [29].

## Results

### An increase in labile Fe2+ level in *A. xylanus* by rapid depletion of oxygen

A previous study demonstrated that the exposure of *A. xylanus* to oxygen stimulates the formation of free flavins and their associated enzymes [5]. Therefore, free flavin‐dependent Fe^3+^ reductase activity was observed in the aerobic cells. However, detecting intracellular Fe^2+^ under oxidizing conditions is difficult because Fe^2+^ is usually oxidized by oxygen. To capture intracellular Fe^2+^, we employed a culture method using a jar fermenter that allowed the rapid switching of the growth environment from aerobic to anaerobic by removing dissolved oxygen with 100% N_2_ gas (sudden anaerobization).

**Fig. 2:**
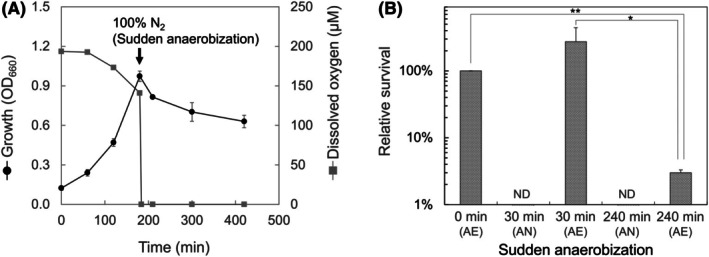
The effect of sudden anaerobization during aerobic growth of *A. xylanus*. (A) The introduction of air into the culture was abruptly cut off by switching 100% N_2_ supply during aerobic growth. The arrow indicates the switching point. The growth of *A. xylanus* was monitored by an optical density of 660 nm (OD_660_). Dissolved oxygen concentrations in the medium were measured using an oxygen electrode. (B) The relative number of viable cells was evaluated following the sudden anaerobization for 0, 30, and 240 min. The cells were plated and incubated aerobically (AE) or anaerobically (AN). All data are presented as the mean values from three biological replicates; error bars indicate the standard deviation (SD). Asterisks indicate significant differences at *P = 0.0087 and **P = 0.00018 according to Student's paired *t*‐test.

Growth inhibition of *A. xylanus* was observed upon sudden anaerobization (Fig. 2A). Oxygen electrode measurements showed that when air in the medium was replaced with N_2_ gas, the dissolved oxygen in the medium was decreased from approximately 140 μm to undetectable levels within a few minutes of introducing 100% N_2_ gas. Monitoring the anaerobic environment for 240 min revealed a slight decrease in OD_660_. Additionally, these cells were unable to form colonies on the agar medium under anaerobic conditions. When the anaerobically cultured cells were re‐exposed to oxygen, the number of viable cells recovered was reduced by 97% at 240 min after the culture switch compared to that at the starting time point (Fig. 2B).

**Fig. 3:**
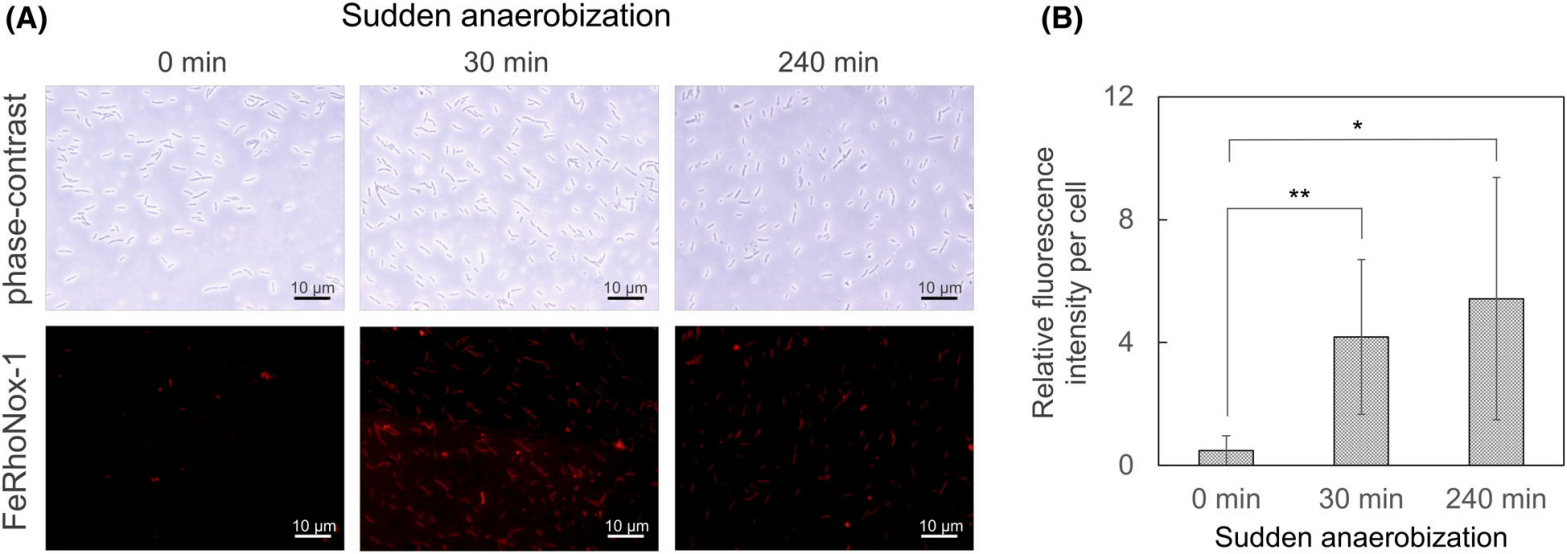
Fluorescence imaging of intracellular Fe^2+^. (A) Representative fluorescence microscopic images of intracellular Fe^2+^ stained with RhoNox‐1 are shown with phase‐contrast images of *A. xylanus* cells which were subject to sudden anaerobization for 0, 30, and 240 min, respectively. The length of scale bars represents 10 μm. (B) Fluorescence intensity of RhoNox‐1 per cell was estimated using the ImageJ software. Data are the mean value from 15 field images obtained from three independent cultures in each condition, and error bar indicates standard deviation. Asterisks indicate significant differences at *P = 0.00025 and **P = 0.000098 according to Student's paired *t*‐test.

Intracellular Fe^2+^ levels were analyzed using RhoNox‐1, a cell‐permeable fluorescent probe that specifically detects labile Fe^2+^ [30]. Labile Fe^2+^ generally refers to Fe^2+^ ions that are weakly protein‐bound or unbound and are considered redox‐active. The cells were incubated with the probe at 0 min (AN0), 30 min (AN30), and 240 min (AN240) after sudden anaerobization. Representative microscopic images of the stained cells are shown in Fig. 3A. The red fluorescence emitted by RhoNox‐1 was more prominent in AN30 and AN240 than in AN0. The mean fluorescence intensity per cell in AN30 and AN240 was 9–11 times higher than that in AN0 (Fig. 3B), indicating that the increase in labile Fe^2+^ was caused by the sudden anaerobization.

### Chemical species in increased Fe^2+^ pool after sudden anaerobization

To further evaluate the increase in labile Fe^2+^ in *A. xylanus*, a whole‐cell ^57^Fe Mössbauer spectroscopy was performed. Prior to the measurement, a switching culture was conducted in the presence of 1 ppm ^57^Fe (III) citrate to allow the cells to assimilate traceable iron isotopes. ^57^Fe‐enriched cells were harvested before (AN0) and after (AN240) sudden anaerobization. Total intracellular iron concentrations were 126 ± 8 μm and 181 ± 3 μm (*n* = 2) in AN0 and AN240, respectively. From these values, the respective absorber thicknesses were estimated to be 0.062 and 0.089 mg/cm^2^, respectively, in terms of native iron.

**Fig. 4:**
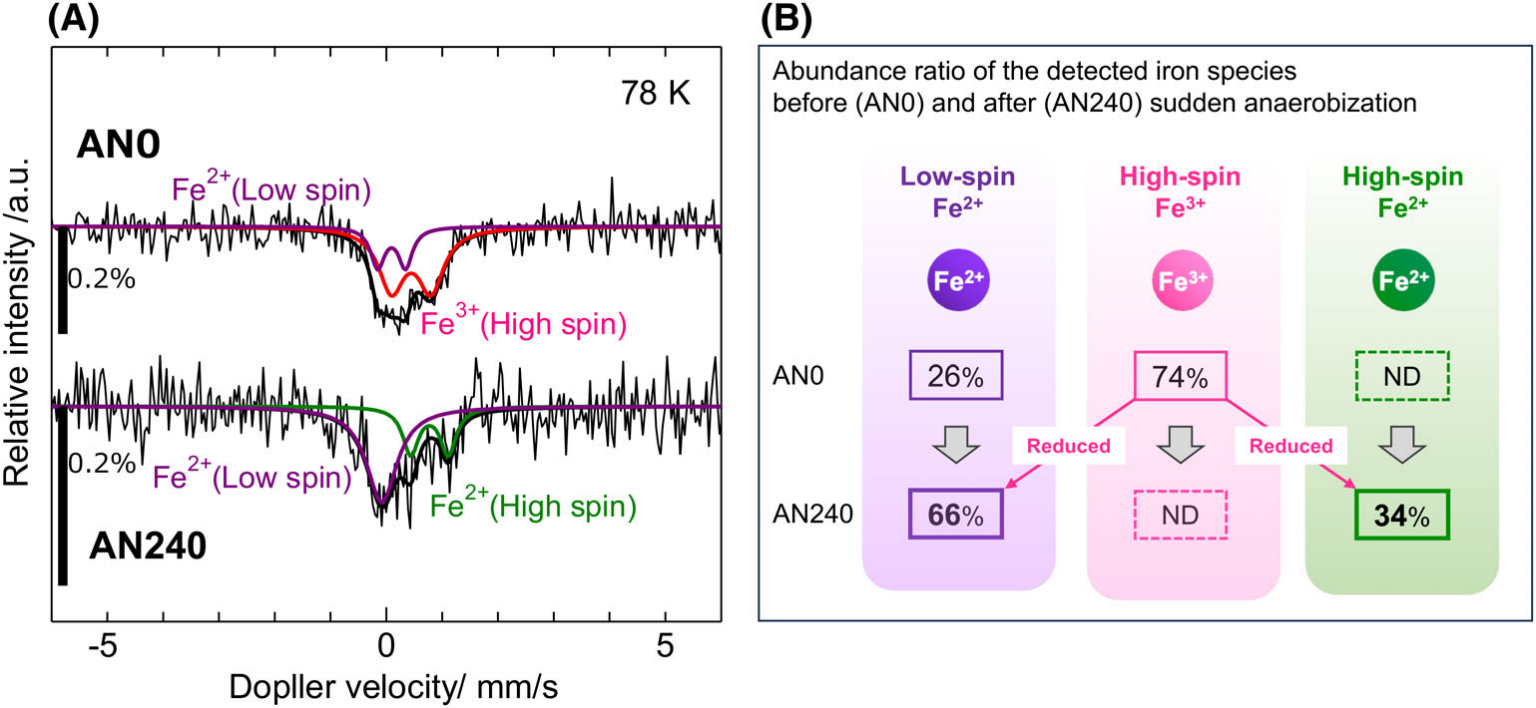
A whole‐cell ^57^Fe Mössbauer analysis in *A. xylanus*. (A) Mössbauer spectrum was obtained at 78 K using *A. xylanus* cells that underwent sudden anaerobization for 0 min (AN0, upper line) or 240 min (AN240, lower line). The length of the black vertical bars is a Mössbauer absorbance‐intensity of 0.2% for each spectrum. (B) Schematic diagram of the variable abundance of iron species detected before (AN0) and after (AN240) sudden anaerobization. The three iron species were assigned by the quadrupole splitting (*ΔE*_Q_) and isomer shift (*δ*) parameters derived from the two Mössbauer spectra (Table 1).

The Mössbauer spectra obtained at 78 K are shown in Fig. 4A. The AN0 spectrum was resolved into two quadrupole doublets. The doublet with the larger isomer shift (IS, *δ*) was assigned to high‐spin Fe^3+^ and the smaller *δ* to low‐spin Fe^2+^ (Table 1). The ratio of the absorption area of Fe^3+^ and Fe^2+^ was 74:26, indicating that, intracellular iron exists mainly in the oxidized form in aerobic cells. In contrast, Fe^3+^ was completely converted to Fe^2+^ in AN240 (Fig. 4B), which is consistent with the results of the Fe^2+^ imaging analysis. The spectrum consisted of two quadrupole doublets due to the two types of Fe^2+^: one low‐spin Fe^2+^ with a smaller *δ* value and the other newly emerged high‐spin Fe^2+^ with a larger *δ* value (Table 1). The absorption area ratio of low‐spin Fe^2+^ to high‐spin Fe^2+^ was 66:34. The quadrupole splitting (QS, *ΔE*_Q_) in combination with IS also provided information regarding the coordination polyhedron [29]. The high‐spin Fe^3+^ and low‐spin Fe^2+^ species were presumed to be octahedral, whereas the high‐spin Fe^2+^ species specifically detected in AN240 could be tentatively assigned to a square‐planar geometry, although the resolution of the obtained spectra was relatively low with an absorption rate of < 0.2%.

**Table 1:** ^57^Fe Mössbauer parameters for *A. xylanus* cells.

Sample	*T* (K)	*δ* (mm/s)	*ΔE*_Q_ (mm/s)	Area (%)	Oxidation state	Spin state
AN0	78	0.45 (3)	0.71 (4)	74 (11)	Fe^3+^	High spin
	78	0.09 (3)	0.50 (5)	26 (8)	Fe^2+^	Low spin
AN240	78	0.77 (4)	0.67 (7)	34 (8)	Fe^2+^	High spin
	78	−0.08 (4)	0	66 (10)	Fe^2+^	Low spin

The values of isomer shift (*δ*) quadrupole splitting (*ΔE*_Q_) were obtained from the fitting results of ^57^Fe Mössbauer measurement for *A. xylanus* (Fig. 4A). Errors in parentheses were obtained from least‐squares fitting of the spectra.

### Free FAD as an efficient electron carrier for reducing Fe^3+^

Oxidation of free reduced flavins by O_2_ competes with Fe^3+^. Therefore, it is possible that more Fe^2+^ is produced by intracellular free flavins under anaerobic conditions than under aerobic conditions. To evaluate free flavin‐dependent ferric reductase activity, the intracellular concentrations of free flavin were measured before and after anaerobization.

Free flavins were extracted from the AN0, AN30, and AN240 cells and fractionated into a soluble fraction including low‐molecular‐weight substances (< 3 kDa). FAD, FMN, and riboflavin were detected in the fractions obtained by HPLC analysis, with FAD being the major free flavin species in all samples (Table 2). The amounts of free FAD in AN0, AN30, and AN240 were 33 ± 1, 22 ± 11, and 11 ± 7 nmol/g dry cell, respectively. Based on the intracellular water volume (5 mL/g dry cell), the intracellular concentrations of free FAD in AN0, AN30, and AN240 cells were estimated to be 7, 4, and 2 μm, respectively.

**Table 2:** Intracellular flavin contents in *A. xylanus*.

Flavins (nmol/g dry cell)		Sample		
		AN0	AN30	AN240
FAD	Total	210 ± 15	186 ± 22	111 ± 17
	Free	33 ± 1	22 ± 11	11 ± 7
FMN	Total	118 ± 15	112 ± 22	82 ± 23
	Free	12 ± 3	10 ± 4	10 ± 4
Riboflavin	Total	31 ± 8	34 ± 8	31 ± 13
	Free	8 ± 4	10 ± 4	9 ± 5

Data are presented as the mean value ± standard deviation (SD) from three independent experiments and expressed in nanomoles of flavin per gram of dry cell.

**Fig. 5:**
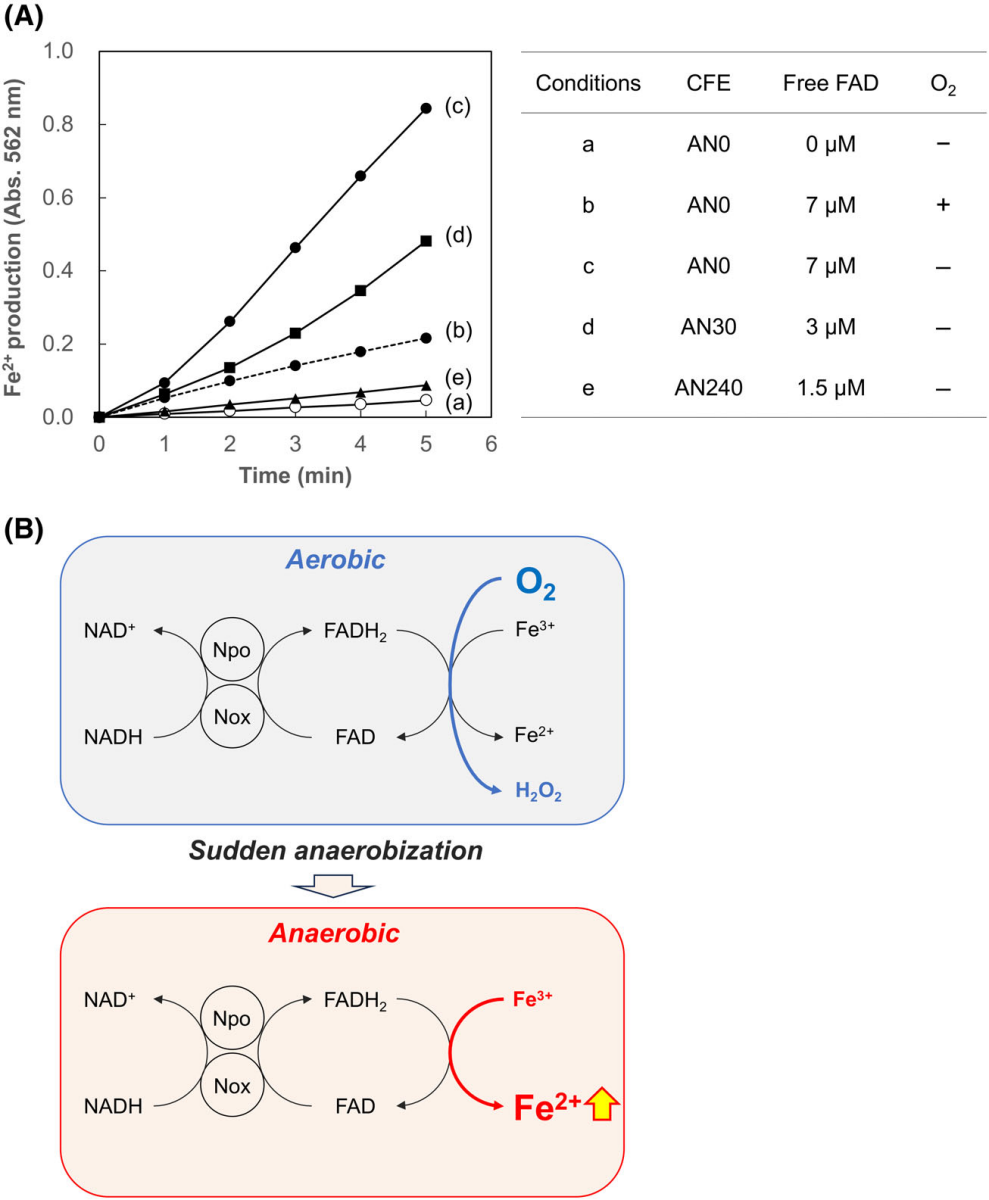
Ferric reductase activity in the presence of free flavin adenine dinucleotide (FAD). (A) Free flavin‐dependent Fe^3+^ reduction was measured using the cell‐free extract (CFE) prepared from cells which were subject to sudden anaerobization for 0 (AN0), 30 (AN30), and 240 min (AN240) under each intracellular concentration of free FAD and the same aeration conditions (with or without oxygen) as the culture experiment (Fig. 2A). These activities were measured spectrophotometrically using ferrozine reagent that forms a purple‐colored complex with Fe^2+^ at 25 °C in the reaction included 50 mm sodium phosphate buffer (pH 7.0), 150 μm NADH, 100 μm ferric citrate, 400 μm ferrozine, CFE containing 200 μg protein, and each intracellular concentration of free FAD. (B) Schematic model of the overproduction of Fe^2+^ via free flavins by sudden anaerobization; free reduced flavins are competitively oxidized by O_2_ and Fe^3+^ substrates under aerobic conditions while they predominantly generate Fe^2+^ under limited O_2_ conditions.

Using cell‐free extracts (CFEs) prepared from AN0, AN30, and AN240 cells, ferric reductase activity was measured at various physiological concentrations of free FAD. NADH was used as an electron donor to generate free reduced flavins, and Fe (III) citrate was added to the assay system as a terminal electron acceptor. An assay using the CFE of AN0 cells showed that free FAD was required for rapid Fe^2+^ generation and electron transfer from NADH to Fe (III) citrate (Fig. 5A). The activity of Fe^2+^ production was higher under anaerobic conditions than aerobic conditions and was also maintained in the CFE of AN30 even when 3 μm free FAD was added in the absence of O_2_. This suggests that the increase in intracellular Fe^2+^ upon sudden anaerobization could be mediated by free flavins (Fig. 5B).

### Aerobic or anaerobic‐specific gene expression after sudden anaerobization

To investigate the growth response to elevated intracellular Fe^2+^ levels, we performed RNA‐Seq analysis of culture‐switched cells. Sudden anaerobization led to significant downregulation of genes involved in flavin synthesis (*ribBA, ribE, ribG*, and *ribH*) and a free flavin‐associated enzyme (*npo*) (Table 3). This observation is consistent with a previous report showing that free flavins and their associated enzymes are expressed under aerobic rather than anaerobic conditions [26]. In contrast, the gene encoding Nox (*nox1*), a major enzyme exhibiting flavin reductase activity, as well as the genes encoding ROS scavenging enzymes (*ahpC* and *sodA*) continued to be expressed after the transition. A comparison of AN0 and AN30 revealed that 94.6% (2280 genes) of all genes showed unchanged expression levels, indicating that *A. xylanus* failed to switch from aerobic to anaerobic metabolism after the environmental shift. Focusing on the two distinct pyruvate metabolic pathways for aerobic and anaerobic growth in central metabolism (Fig. 1), the anaerobic‐specific genes such as those encoding pyruvate formate‐lyase (*pflB*) and aldehyde dehydrogenase (*adh*) were not induced. However, corresponding genes in the aerobic pathway, encoding the pyruvate dehydrogenase complex (*pdhA, pdhB, pdhC*, and *pdhD*) and Nox (*nox1*), were expressed. This suggests that during anaerobiosis, Nox receives electrons from NADH generated through the aerobic glycolytic and pyruvate metabolic pathways and mediates Fe^3+^ reduction.

**Table 3:** Comparative RNA‐Seq analysis before and after sudden anaerobization.

Genes	Products	Fold changes		DEGs	
		AN0 vs AN30	AN0 vs AN240	AN0 vs AN30	AN0 vs AN240
Glycolysis					
*pgi*	glucose‐6‐phosphate isomerase	−2.3	−1.7	n.s.	n.s.
*pfkA*	6‐phosphofructokinase	−1.1	−1.5	n.s.	n.s.
*fbaA*	fructose‐bisphosphate aldolase	−1.6	−1.6	n.s.	n.s.
*gapA*	glyceraldehyde 3‐phosphate dehydrogenase	−1.1	1.0	n.s.	n.s.
*pgk*	phosphoglycerate kinase	−1.2	−1.2	n.s.	n.s.
*gpmI*	2,3‐bisphosphoglycerate‐independent phosphoglycerate mutase	−1.8	1.1	n.s.	n.s.
*eno*	enolase	−2.2	1.1	n.s.	n.s.
*pykA*	pyruvate kinase	−2.2	−1.9	n.s.	n.s.
Pyruvate metabolisms					
(a) common to aerobic and anaerobic pathways					
*pta*	phosphate acetyltransferase	1.6	1.0	n.s.	n.s.
*ackA*	acetate kinase	−1.4	−1.1	n.s.	n.s.
*acyP*	acylphosphatase	1.4	−1.4	n.s.	n.s.
(b) specific for aerobic pathway					
*pdhA*	pyruvate dehydrogenase complex E1 component alpha subunit	1.2	−2.1	n.s.	n.s.
*pdhB*	pyruvate dehydrogenase complex E1 component beta subunit	−1.0	−1.8	n.s.	n.s.
*pdhC*	pyruvate dehydrogenase complex E2 component	−1.2	−1.5	n.s.	n.s.
*pdhD*	pyruvate dehydrogenase complex E3 component	−1.5	−1.3	n.s.	n.s.
(c) specific for anaerobic pathway					
*pflB*	pyruvate formate‐lyase	1.4	−1.1	n.s.	n.s.
*adh*	alcohol dehydrogenase	1.7	1.3	n.s.	n.s.
*adhE*	acetaldehyde/alcohol dehydrogenase	1.4	1.4	n.s.	n.s.
*aldH*	aldehyde dehydrogenase	−1.1	1.1	n.s.	n.s.
Oxygen metabolic enzymes					
*nox1*	NADH oxidase	−2.5	−2.4	n.s.	n.s.
*npo*	NAD(P)H oxidoreductase	−6.7	−4.3	Down	Down
*ahpC*	alkyl hydroperoxide reductase C	−2.6	−2.5	n.s.	n.s.
*sodA*	manganese superoxide dismutase	−1.0	−1.0	n.s.	n.s.
Flavin synthesis					
*ribBA*	3,4‐dihydroxy‐2‐butanone 4‐phosphate synthase/GTP cyclohydrolase II	−13.6	−15.7	Down	Down
*ribE*	riboflavin synthase alpha chain	−12.9	−14.4	Down	Down
*ribG*	diaminohydroxyphosphoribosylaminopyrimidine deaminase/5‐amino‐6‐(5‐phosphoribosylamino) uracil reductase	−10.3	−11.2	Down	Down
*ribH*	6,7‐dimethyl‐8‐ribityllumazine synthase	−17.3	−20.1	Down	Down
*ribC*	FAD synthase/riboflavin kinase	1.1	1.2	n.s.	n.s.

Transcripts per million (TPM)‐normalized expression levels were calculated using three independent RNA‐Seq analyses. Differentially expressed genes (DEGs) were defined as genes showing a fold change ≥ |2| and false discovery rate (FDR) *P*‐value of < 0.05. ‘n.s.’ indicates not significant (not DEGs).

## Discussion

Free flavin‐dependent ferric reductases are present in many microorganisms [5, 12, 13, 14, 15, 16, 17, 18, 19, 20, 21, 22]. In addition, the Fe^2+^‐generating activity of these flavin reductases may promote the production of hydroxyl radicals via the Fenton reaction [17, 18, 19, 20, 31]. We previously reported that *A. xylanus* contains free flavins that enhance Fe^2+^ production during aerobic growth [5]. However, whether free reduced flavins can produce redox‐active labile Fe^2+^ in cells remains unclear.

In this study, we demonstrated that a rapid shift to anaerobic conditions during the aerobic growth of *A. xylanus* led to an increase in intracellular labile Fe^2+^ levels, as monitored using the specific fluorescent probe RhoNox‐1, which specifically detects Fe^2+^ ions [30] (Fig. 2). Furthermore, Fe^2+^ production activity in the CFE was enhanced in the presence of physiological concentrations of free FAD under anaerobic conditions rather than under aerobic conditions, mimicking the above observation (Fig. 5). This enhancement may be due to the low redox potential of free reduced flavins, whose oxidation by Fe^3+^ competes with O_2_ [6, 7, 8]. The two flavoproteins, NADH oxidase (Nox) and NAD(P)H oxidoreductase (Npo), have been identified in *A. xylanus* as flavin reductases capable of reducing intracellular concentrations of free FAD (3–7 μm) with *K*_m_ values of 37 and 71 μm, respectively [5]. These results suggest that intracellular free flavins are involved in the generation of labile Fe^2+^ in *A. xylanus*.

The increase in intracellular Fe^2+^ due to sudden anaerobization was confirmed by whole‐cell ^57^Fe Mössbauer analysis, which suggested that the low‐spin Fe^2+^ and high‐spin Fe^2+^ species were produced from high‐spin Fe^3+^ species (Fig. 4). This suggests that the labile Fe^2+^ pool consists of multiple Fe^2+^ species with varying chemical characteristics. Low‐spin Fe^2+^ species were observed under both aerobic and anaerobic conditions, whereas high‐spin Fe^2+^ species were detected only under anaerobic conditions, suggesting that the latter may be oxygen‐sensitive. Based on ligand‐ and crystal‐field theories, low‐spin Fe^2+^ species may be coordinated by strong field ligands such as carbon and nitrogen, whereas high‐spin Fe^2+^ species may be coordinated by weak field ligands such as oxygen and sulfur. However, specific Fe^2+^ metabolites could not be identified due to a lack of reference Mössbauer spectra. Previous studies on whole‐cell ^57^Fe Mössbauer of *E. coli* have suggested the presence of several Fe^2+^ metabolites, such as ferritin [32], oligomeric ferrous sugar phosphate [33], Fe‐citrate, and Fe‐ATP [34]; however, none of these Mössbauer spectra match those of the two Fe^2+^ species found in *A. xylanus*. In addition, *A. xylanus* is not considered a source of haem iron because it lacks haem synthesis genes [26].

In this study, we tentatively assigned the high‐spin Fe^2+^ species found in *A. xylanus* cells to square‐planar high‐spin Fe^2+^ (Fig. 4A). To our knowledge, this atypical Fe^2+^ state has not been previously observed as labile iron in biological samples and may result from the unique physiology of *A. xylanus*, namely, Fe^2+^ production via an intracellular free flavin‐associated system. However, these Fe^2+^ species were assigned by conservative deconvolution analysis to prevent overinterpretation, because the low iron concentration in the cultured cells limited the quality of the Mössbauer spectra. Further studies aimed at improving the spectral quality with high‐resolution measurements are required to clearly identify the chemical nature of this Fe^2+^ species and to compare its biological functions with those of common Fe^2+^ species. For the high‐spin Fe^3+^ species observed in aerobic cells, the *δ* and*ΔE*_Q_ values were similar to those of Fe (III) citrate [32]. This is consistent with the culture conditions, under which *A. xylanus* was grown in the presence of ^57^Fe (III) citrate during sample preparation.

**Fig. 6:**
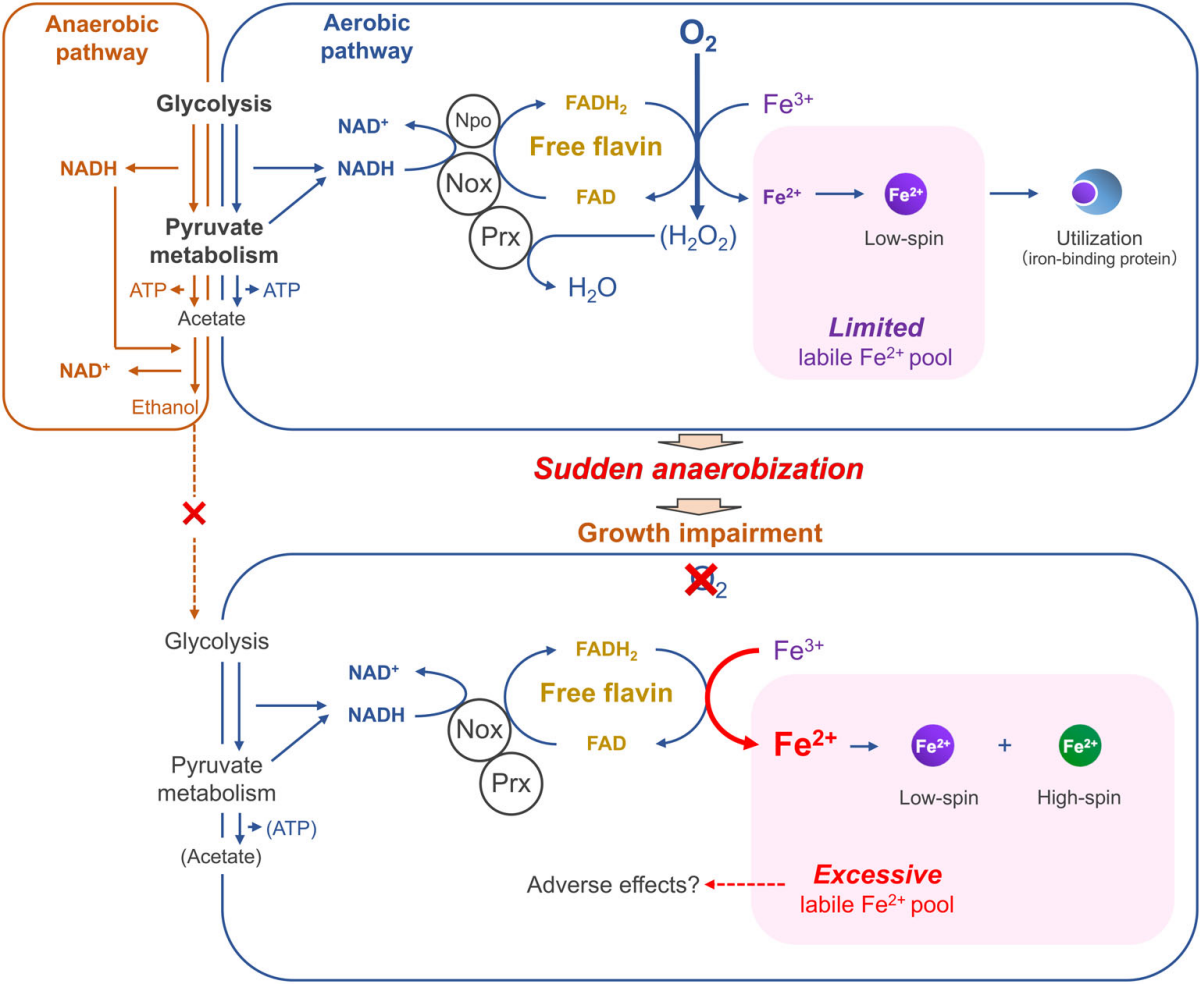
A proposed model for excessive labile Fe^2+^ production via free flavins upon sudden anaerobization in *A. xylanus*. During aerobic growth, the oxidation of free reduced flavins with O_2_ maintains labile Fe^2+^ production at an adequate level for biological iron utilization (upper panel). However, when O_2_ is suddenly removed, the production of labile Fe^2+^ accelerates, and excess labile Fe^2+^ accumulates, causing growth impairment (lower panel).

During the aerobic growth of *A. xylanus*, O_2_ and Fe^3+^ reduction systems associated with free flavins can simultaneously generate H_2_O_2_ and Fe^2+^ (Fig. 1). Although this system may promote the generation of hydroxyl radicals via the Fenton reaction, the ability of *A*. *xylanus* to sustain aerobic growth suggests that it can control Fe^2+^‐associated oxidative stress [25, 26]. The present study showed that Fe^2+^ was less abundant than Fe^3+^ in aerobic cells, resulting in a relatively small labile Fe^2+^ pool (Figs 3 and 4). Living cells of *A. xylanus* exhibit robust oxygen consumption, which is attributed to the reduction of O_2_ by free intracellular flavins [5]. Consequently, the excessive production of labile Fe^2+^ appeared to be suppressed by the active oxidation of free reduced flavins by O_2_ during aerobic growth, a mechanism supported by enzymatic assays (Fig. 5). This behavior of free flavins may contribute to maintaining the intracellular labile Fe^2+^ pool at a moderate level, which is sufficient for essential cellular functions while minimizing damage caused by oxidative stress. *A. xylanus* requires iron for aerobic growth, and several genes encoding iron‐associated proteins are expressed in the presence of oxygen [5, 26]. These proteins require the incorporation of labile Fe^2+^ into their catalytic center to facilitate aerobic metabolism [16]. Aerobic growth can produce large amounts of H_2_O_2_, which can be efficiently reduced to water using the Nox‐Prx system. The catalytic efficiency of this system is much higher than that of O_2_ reduction by Nox in the presence of 8 μM free FAD [5, 24]. Therefore, free flavins and their associated enzymes may play roles in oxygen detoxification and in regulating and providing Fe^2+^ for biological utilization in *A. xylanus* [5] (Fig. 6). This model aligns with a recently proposed concept for bacteria capable of extracellular electron transfer, in which extracellular free flavins facilitate oxygen detoxification while simultaneously reducing soluble iron under oxic conditions [35].

Sudden anaerobization significantly inhibited the growth of *A. xylanus*, resulting in the accumulation of large amounts of labile Fe^2+^. A similar growth impairment associated with increased Fe^2+^ due to free flavins has also been reported in *E. coli*, in which respiration was blocked by cyanide [31]. However, there is a critical difference between the two observations; *E. coli* with a blocked respiratory system promoted the production of toxic hydroxyl radicals upon exposure to exogenously added H_2_O_2_. In contrast, growth impairment of *A. xylanus* occurred under anaerobic conditions, even without exposure to H_2_O_2_.

Growth failure may be caused by a redox imbalance. RNA‐Seq analysis indicated that *A. xylanus* was unable to switch pyruvate metabolism from the aerobic to anaerobic pathways after sudden anaerobization (Table 3). Specifically, no significant changes were observed in the gene expression of Nox and alcohol dehydrogenase (ADH), which are functionally identical enzymes that catalyze the oxidation of NADH during aerobic and anaerobic pyruvate metabolism, respectively, to regenerate NAD+, which is required for glycolysis (Fig. 1). Nox oxidizes NADH via free FAD using O_2_ or Fe^3+^ as the terminal electron acceptors. Consequently, the cells were unable to regenerate NAD^+^ under the intentionally created O_2_‐ and Fe^3+^‐limited conditions (Figs 2 and 4). This maladaptive gene expression may disturb glycolytic and pyruvate metabolism during anaerobiosis, ultimately leading to ATP insufficiency (Fig. 6). Recent studies have suggested that labile Fe^2+^ may be a determinant that controls the epigenome and alters gene expression [36]. However, whether the excessive accumulation of labile Fe^2+^ contributes to transcriptional impairment in *A. xylanus* remains unclear.

Labile Fe^2+^ can drive the Fenton‐type chemistry and react with H_2_O_2_ and other cellular materials to generate toxic radicals. For example, lipid hydroperoxides (LOOH) are reductively cleaved by Fe^2+^ to form highly reactive alkoxyl radicals (LO^•^) [37]. Nitrite (NO_2_^−^) and nitrate (NO_3_^−^) can also react with Fe^2+^ to generate reactive nitrogen species including nitrite oxide (NO) [38]. Because ROS generation was not assessed in this study, it is unclear whether the growth impairment observed in *A. xylanus* is directly associated with these radical species. However, interestingly, recent studies have reported that RhoNox‐1, which was used for labile Fe^2+^ imaging in the present study, inhibits ferroptosis, an iron‐dependent nonapoptotic cell death process, through the oxidation of Fe^2+^ in human cells [39, 40]. Therefore, further studies are required to elucidate the cellular targets of labile Fe^2+^ under physiological conditions in *A. xylanus* to better understand the biological roles of free flavins and iron toxicity.

Our findings suggest that the production of labile Fe^2+^ via free flavins is regulated by molecular oxygen, allowing safe iron utilization during the aerobic growth of *A. xylanus*. However, we acknowledge that direct *in vivo* evidence for the active involvement of free flavins in Fe^2+^ production remains to be fully established. To address this, further experiments, such as modulating cellular free flavin availability, are required. Furthermore, evaluating the potential cytotoxicity of labile Fe^2+^ during the aerobic growth of *A. xylanus* represents an important subject for future studies to support our present conclusions.

## Conclusion

We propose that intracellular free reduced flavins are involved in the reduction of Fe^3+^ to generate labile Fe^2+^ in *A. xylanus*. This process is thought to be controlled by the oxidation of free reduced flavins by molecular oxygen, thereby ensuring safe iron utilization during aerobic growth.

## Conflicts of interest

The authors declare no conflicts of interest.

## Author contributions

SK and YN conceived and designed the study. SK, YS, and TK acquired and analyzed the data. YE, KK, NT, TT, MI, HI, KN, TS, MU, and AA provided critical advice. SK and YN drafted the manuscript. All the authors reviewed the manuscript and approved the final version.

## Supporting information


**Fig. S1.** The effect of sudden oxygen exposure during anaerobic growth. Growth curve of *A. xylanus* was shown, in which air (21% O_2_) was introduced into the anaerobic culture once the optical density of 660 nm (OD_600_) reached approximately 1.0. Adaptation to the sudden oxygen exposure was observed in three independent experiments.

## Data Availability

The data supporting the findings of this study are available from the corresponding author upon request.
