# Supplementary material for: Sudden anaerobization in Amphibacillus xylanus increases intracellular labile ferrous iron and inhibits cell growth
Source: FEBS Open Bio. 2026 Apr 27;16(10):1911–24. doi: 10.1002/2211-5463.70255 (PMC13398963; doi:10.1002/2211-5463.70255)
Supplement: Supplementary file 1 [file FEB4-16-1911-s001.docx]

**Fig. S1.** The effect of sudden oxygen exposure during anaerobic growth. Growth curve of *A. xylanus* was shown, in which air (21% O_2_) was introduced into the anaerobic culture once the optical density of 660 nm (OD_600_) reached approximately 1.0. Adaptation to the sudden oxygen exposure was observed in three independent experiments.
